# Gamification Integration in Technological Devices for Motor Rehabilitation in Parkinson Disease: Scoping Review

**DOI:** 10.2196/69433

**Published:** 2025-07-04

**Authors:** Pere Bosch-Barceló, Oriol Martínez-Navarro, Maria Masbernat-Almenara, Carlos Tersa-Miralles, Anni Pakarinen, Helena Fernández-Lago

**Affiliations:** 1 Department of Nursing and Physiotherapy, Universitat de Lleida, C/ Montserrat Roig, nº2, Lleida, 25198, Spain, 34 973702430; 2 Health Care Research Group (GReCS), Lleida Institute for Biomedical Research Dr Pifarré Foundation (IRBLleida), Lleida, Spain; 3 Department of Nursing Science, University of Turku, Turku, Finland; 4 Consolidated Research Group: Society, Health, Education and Culture (GESEC), Department of Nursing and Physiotherapy, Universitat de Lleida, Lleida, Spain

**Keywords:** gamification, motor rehabilitation, Parkinson disease, rehabilitation, physiotherapy

## Abstract

**Background:**

Parkinson disease (PD) is a rapidly growing neurological condition worldwide. While physiotherapy and exercise are effective interventions, the addition of motivational aspects that improve adherence could be beneficial for people with PD. Incorporating technological devices into motor rehabilitation, coupled with gamification elements, could enhance the relevance of rehabilitation and alleviate motor symptoms.

**Objective:**

The aim of this scoping review was to identify and classify the technological devices that integrate gamification elements used in motor rehabilitation in PD, and to describe the justification behind the use of these devices and elements in this context.

**Methods:**

We conducted a scoping review following the framework proposed by Joanna Briggs Institute, along with the PRISMA-ScR (Preferred Reporting Items for Systematic Reviews and Meta-Analyses Extension for Scoping Reviews) guidelines. Major health science databases (MEDLINE, EMBASE, Scopus, Cochrane, Web of Science, PsycINFO, and Epistemonikos) were systematically searched. Relevant studies were included if they used technological interventions with gamification elements for motor symptom rehabilitation in PD. Gamification elements were extracted and categorized based on established frameworks, and content analysis was used to review the justifications for the use of technologies integrating gamification.

**Results:**

A total of 4451 studies were retrieved from the search. After the abstract and full-text screening, 81 studies were eligible for data extraction. The analysis identified 453 gamification elements across studies, with development and accomplishment being the most prominent core drive. Progress/feedback was the most frequently used element (79/81, 98% of studies), followed by points (70/81, 86%) and levels/progression (66/81, 81%). Other notable elements included badges, leaderboards, and customization, while several core drives, like ownership and possession, lacked reported elements. Most interventions were delivered through commercial video game consoles (33/81, 41%), followed by computer-based systems (32/81, 40%). Tablet-based applications and integrated rehabilitation platforms were used in 11% (9/81) and 10% (8/81) of the studies, respectively. The expected roles of technology were clear, but intentional use of gamification was scarce.

**Conclusions:**

This scoping review highlights the widespread adoption of technologies integrating gamification elements for motor symptom rehabilitation in individuals with PD. However, it also underscores a critical gap in understanding and justifying gamification mechanics. The current landscape relies heavily on commercial video games and emphasizes performance-based experiences, lacking theoretical grounding.

## Introduction

Parkinson disease (PD) is the second most prevalent neurodegenerative disease in the world, as well as the fastest-growing one [1,2]. Over the last 20 years, the global impact of PD in terms of deaths and disability has increased by more than twofold [3]. As of 2021, nearly 12 million people around the world were living with PD, and this number is expected to increase by approximately 50% by 2035 [4].

PD is mainly characterized by the presence of several cardinal motor symptoms, mainly stiffness, bradykinesia, tremor, and postural instability [5]. The progression of these symptoms heavily impacts the ability of people with PD to manage daily activities [6]. Pharmacological treatment options for PD motor symptoms are mainly comprised of dopamine-based options: levodopa, dopamine agonists, and monoamine oxidase-B inhibitors [7]. However, these pharmacological choices come with their own array of issues, for example, Levodopa is known to eventually cause undesirable and involuntary movements known as dyskinesias in over half of patients after 6 years [8]. Due to these secondary effects, other treatment options are needed for people with PD. Physical therapy and exercise training have proven capable of inducing long-term improvements in motor symptoms and physical function among individuals with PD [9]. These interventions may also enhance the efficacy of pharmacological treatments, and help delay disease progression and age-related decline, with certain modalities additionally improving quality of life, thus becoming a crucial component in the management of people with PD [9,10].

In the last decades, a wide array of technological devices has been incorporated into the rehabilitation of neurological conditions to assist training. In this context, options such as virtual reality (VR) [11,12], robotics [13], smartphone apps [14], telerehabilitation options [15], and videogames and exergames [16] have been studied and shown positive effects on people with PD. This massive implementation of technological solutions often follows the implication that, in trying to affect and improve the users’ experience and behavior, mechanics reminiscent of games are used. This concept of “the use of game design elements in non-game contexts” is widely known as gamification [17]. In the context of rehabilitation, the integration of gamification elements into technological devices is designed to create a more engaging and challenging experience for users. This approach is anticipated to enhance adherence and motivation among participants [18]. The combination of gamification and VR could even add benefits for older people in physical and cognitive domains while allowing complex training scenarios such as dual task training to be performed [19,20].

To effectively incorporate gamification elements across various intervention designs, it is essential to thoroughly understand gamification principles and their interaction with human motivation. Frameworks play a pivotal role in organizing available gamification elements into coherent categories, thereby guiding developers in selecting the most appropriate elements to achieve the desired user experience. Common frameworks include the Octalysis Framework by Kai [21], which identifies 8 core drives of human motivation, such as epic meaning and social influence; and the Marczewski 52 Gamification Mechanics and Elements [22], which classify elements based on the type of player they best cater to. However, the decision of which gamification elements to apply in interventions is often not thoroughly considered, in a context where treatment options should cater to the needs of end users [23]. Recent studies point to most VR rehabilitation options for PD being administered through commercial videogames, which are designed for entertainment in healthy individuals, far away from the context of rehabilitation for people with PD [24-26].

In PD, where apathy and depression are often present, long-term adherence to exercise and rehabilitation is concerning [27,28]. Previous research has shown that the implementation of VR does not warrant a higher adherence to treatment [11]. Strategies to promote motivation to stay involved in the rehabilitation process are key to long-term success in a population with a tendency to drop out [29]. Gamification, by integrating elements like feedback, rewards, and goal setting, may help sustain engagement and counteract the apathy and depression common in PD, thereby supporting long-term adherence to rehabilitation. However, current literature lacks information on the implementation of gamification in technological devices aimed at rehabilitating motor conditions in PD. Understanding which elements are currently used and the types of technological solutions they are integrated into is essential for developing further interventions that address the specific needs of individuals with PD. Therefore, the aim of this scoping review is to identify and classify the gamification elements and technologies used in motor rehabilitation in PD and to describe the justification behind the use of gamification and technology in this context. Specifically, this review focuses on the classification of gamification mechanics available, the types of technological devices used, and the motor symptoms these interventions target. Additionally, it explores the justifications used by each study for the inclusion of gamification elements and technological devices for motor rehabilitation in PD.

## Methods

### Registration and Framework

This Scoping Review is registered at the Open Science Framework database (OSF.IO/tx3d9). This review followed the Joanna Briggs Institute methodology for scoping reviews [30] and also adhered to the PRISMA-ScR (Preferred Reporting Items for Systematic Reviews and Meta-Analyses Extension for Scoping Reviews) to ensure quality, transparency and reproducibility [31].

### Objective

The objective of this study is to explore the use of technological devices integrating gamification in the rehabilitation of motor symptoms for individuals with PD. Specifically, it aims to identify the gamification elements and technological devices used in this context, classify the gamification elements based on the core motivational drives they target and the devices according to the motor symptoms they address, and describe the rationale behind their application in enhancing motor symptom rehabilitation for people with PD.

### Eligibility Criteria

All peer-reviewed articles and peer-reviewed conference papers published in English or Spanish were included. Gray literature sources were excluded to maintain methodological consistency, ensuring all included studies underwent formal peer evaluation and were fully accessible. To guide the eligibility criteria, the Population, Concept, and Context framework was used, in which population, concept, and context are defined to guide study selection [30]. Population refers to people with PD, with no restrictions applied regarding specific subpopulations, gender, disease stage, or geographical location. The Concept covered intervention studies that described motor rehabilitation through technological solutions incorporating clearly identifiable gamification elements. Studies that used commercial video games originally developed for entertainment were also included. While Deterding’s [17] definition of gamification refers to the use of game elements in nongame contexts, implying the exclusion of full video games aimed at entertainment, the therapeutic application of these games in rehabilitation effectively shifts their context. As a result, built-in motivational features such as points, progression systems, and feedback mechanics can be considered relevant gamification elements when used for rehabilitation in PD. In situations where not enough details were given regarding the intervention to chart its gamification elements, the studies were excluded. No restrictions regarding Context or year of publication were established. Articles that met the selection criteria were incorporated without assessing their quality, as the objective of scoping reviews is to chart the pertinent works in a particular area and pinpoint areas lacking research [30].

### Information Sources and Search Strategy

The search strategy was developed and conducted by a librarian on November 23, 2023, across 7 electronic databases: MEDLINE, EMBASE, Scopus, Cochrane, Web of Science, PsycINFO, and Epistemonikos. A combination of controlled vocabulary and free-text keywords was used to ensure retrieval of all potentially relevant studies. The search focused on terms related to PD, gamification, rehabilitation, motor symptoms, and various technological rehabilitation strategies, such as VR, videogames, and exergames. Boolean operators were applied to refine the search, and search strings were adapted to match the indexing structures of each database. The search strategy used for each database is detailed in Multimedia Appendix 1. No systematic or scoping reviews on the specified topic were identified in the Joanna Briggs Institute Systematic Review Register, PROSPERO, or biomedical and nursing databases.

### Study Selection and Data Extraction

All articles extracted from databases were imported to Covidence [32] and checked for duplicates by using its duplicate detection system. Given the large volume of references obtained, the initial title and abstract screening were independently conducted by 3 reviewers (PBB, OMN, and MMA) rather than 2, as initially proposed in the registered protocol. Any disagreements during this screening stage were resolved through consensus discussions involving all 3 reviewers. In the next step, full-text screening was independently performed by the same 3 reviewers, and inconsistencies were again resolved by consensus. Data extraction of all included articles was conducted by PBB.

A total of 4451 articles were retrieved from all databases. After duplicates were removed, both by Covidence and manually, 48.48% (2158/4451) of all articles underwent title and abstract reviewing. After this first title and abstract screening, 87.62% (1891/2158) of articles were excluded. A total of 267 articles were left for full-text screening, 69.66% (186/267) of which were excluded.

### Synthesis of Results

General study information such as location, sample size, age, disease duration, gender, year, and study design were collected. Specific data pertinent to the review’s objectives was also collected.

In order to identify and name the gamification elements found in each study, the Marczewski 52 Gamification Mechanics [22] nomenclature was used due to its clear terminology and ease of use. All gamification elements identified were then classified using the Kai Octalysis Framework [21] due to its focus on motivation and end user interaction with the system. The Octalysis framework delineates human motivation into 8 different core drives: epic meaning and calling, development and accomplishment, empowerment of creativity and feedback, ownership and possession, social influence and relatedness, scarcity and impatience, unpredictability and curiosity, and loss and avoidance. All gamification elements identified using Marczewski’s framework [22] were classified with the core drive they most effectively addressed.

Technological devices integrating gamification were classified based on the type of platform they were used with and also based on which symptoms they intended to address.

Finally, a qualitative content analysis using an inductive approach was conducted to identify the justifications used by each study for the inclusion of gamification elements and technological devices for motor rehabilitation in PD [33]. Data from all included studies were coded and organized into categories, which were then clustered into broader themes with the support of a hierarchical tree diagram. Definitions were developed for each code, category, and subcategory. Two reviewers (PBB and HFL) carried out the analysis independently using Excel, and then discussed how to group codes, align interpretations, and resolve discrepancies. Final themes were reviewed and refined through team-based triangulation to ensure consistency and credibility.

## Results

### General Information

**Figure 1.**
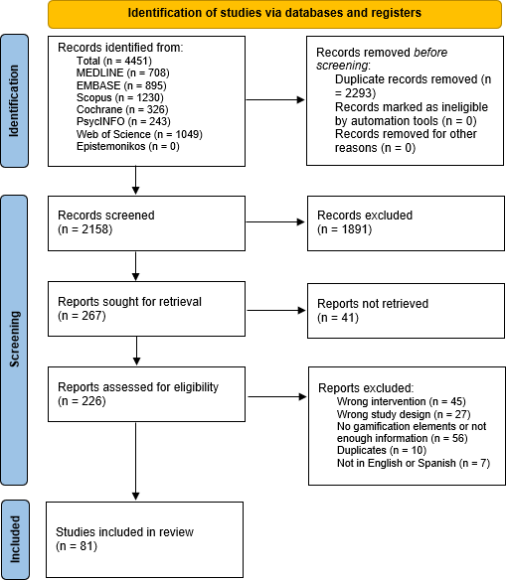
PRISMA (Preferred Reporting Items for Systematic Reviews and Meta-Analyses) flow diagram.

Altogether 81 studies were included in the final review (Multimedia Appendix 2) [34-114]. The PRISMA flow diagram (Figure 1) [115] details the steps followed during the screening process and data on number of excluded studies as well as reasons for exclusion in the full-text step. Of the 81 articles included, over 50% (41/81) were published in the year 2019 or later. Italy and Brazil were the 2 countries where most research was produced, with 18.5% (14/81) and 17.3% (13/81), respectively. The most frequent study design consisted of randomized controlled trials, which accounted for 49% (40/81) of all studies, followed by quasi-experimental studies, which accounted for 46% (37/81) of all studies. Sample sizes from the studies included ranged from 2 to 302 participants, with an average sample size of 32.2 participants. The mean age for participants was 66.64 (SD 5.05) years, with a mean of 2.29 (SD 0.57) on the Hoehn and Yahr [116] scale for disease severity and a mean disease duration of 7.35 (SD 2.70) years. A total of 39.39% of all participants were female. Most studies (62/81, 77%) implemented their interventions in an in-person format, while 23% (19/81) used telerehabilitation. Additionally, 1% (1/81) of studies used a hybrid approach, combining both in-person and remote intervention formats.

### Identification and Classification of Gamification Elements and Technological Devices

#### Gamification Elements

Detailed data on the frequency and distribution of the identified gamification elements and their association with the Octalysis Framework’s 8 Core Drives of motivation can be found in Table 1.

**Table 1.** Distribution of use of gamification elements and classification according to the Octalysis Framework.

Octalysis’ core drive and gamification elements	Times used (out of 81 studies)	Percentage of total studies where used	Percentage of total used elements
Epic meaning and calling			
Quests [59,60]	2	2	<1
Development and accomplishment			
Progress/Feedback [34-86,88-110,112-114]	79	98	17
Points [34-40,42-44,47,50-52,54-58,62-71,73-107,109-114]	70	86	15
Levels/Progression [34-39,41-45,47,50-52,54,56,58,61-80,82-84,86-89,91-97,99-103,106-114]	66	81	15
Badges [35-38,51,52,54,56,58,62,64,66,67,70,71,73,77-79,82,86,90,92,93,96,97,99-101,114]	30	37	7
Leaderboards [36-38,51,52,54,56,58,62,64,66,67,70,71,73-75,77-79,82,88,91,95-97,99-101,114]	29	36	6
Challenges [36-38,51,52,54,56,58,62,64,66,67,70,71,73,77-79,82,91,96,97,99-101,114]	26	32	6
Empowerment of creativity and feedback			
Customization [36-38,45,51,52,54,56,58,62,64,66,67,70,71,73-75,77-79,82,86,91-93,96,97,99-101,114]	32	40	7
Ownership and possession			
—^a^	—	—	—
Social influence and relatedness			
Competition [36-38,51,52,54,56,58,62,64,66,67,70,71,73,77-79,82,86,91-93,96,97,99-101,114]	29	36	6
Knowledge share [59,60]	2	2	<1
Guilds/Teams [59,60]	2	2	<1
Scarcity and impatience			
Unlockables [50]	1	1	<1
Unpredictability and curiosity			
—	—	—	—
Loss and avoidance			
Consequences [36-38,47,52,54,56,58,62,64,66,67,70,71,73,77,79,82,88,91,96,97,99,101,107,109,110,113,114]	30	37	7
Time Pressure [35-38,46,48,49,51,52,54,56,58,62,64-67,70-73,77-79,82,86,91-93,96,97,99-101,109,110,114]	37	46	8

a Not available.

A total of 453 gamification elements were identified across all included studies, categorized into 14 distinct types based on the Marczewski classification of gamification mechanics [22]. For the epic meaning and calling core drive, only quests were identified as a relevant gamification element, appearing 2 times across studies. This element was used in 2.47% (2/81) of the studies, accounting for 0.44% (2/4453) of the total gamification elements used. The development and accomplishment core drive was the most prominent category, containing a variety of gamification elements and showing extensive use across the studies. Progress/feedback mechanics were the most frequently used element, appearing in 79 studies (97.53% of studies) and representing 17.44% (80/453) of the total gamification elements used. Other commonly used elements included points, which appeared in 86.42% (70/81) of studies and accounted for 15.45% (70/453) of total elements; levels/progression, which appeared in 81.48% (66/81) of studies and represented 14.57% (66/453) of total elements; badges, used in 37.04% (30/81) of studies, accounting for 6.62% (30/453) of total elements; leaderboards, used in 35.80% (29/81) of studies, making up 6.40% (29/453) of total elements; and challenges, which appeared in 32.09% (26/81) of studies and represented 5.72% (26/453) of total elements. The empowerment of creativity and feedback core drive included only one gamification element, customization, which was used in 32 studies (39.51% of studies) and accounted for 7.06% (32/453) of total elements. Neither the ownership and possession core drive nor the unpredictability and curiosity drive showed any specific gamification elements reported across the studies. Under the social influence and relatedness core drive, elements like competition, knowledge share, and guilds/teams were identified. Competition was used in 35.80% (29/81) of studies, contributing 6.40% (29/81) to the total elements, while both knowledge share and guilds/teams appeared only in 2.47% (2/81) of studies, accounting for a mere 0.44% (2/453) of total elements each. The scarcity and impatience drive included only one element, unlockables, which was used in just one study, appearing in 1.23% (1/81) of studies and making up only 0.22% (1/453) of the total gamification elements. Last, the loss and avoidance core drive included the elements “consequences” and “time pressure”. Consequences were used in 37.04% (30/81) of studies, contributing 6.62% (30/453) to the total elements, while time pressure appeared in 45.68% (37/81) of studies, making up 8.14% (37/453) of the total elements used.

#### Technological Devices Integrating Gamification

##### Overview

**Figure 2.**
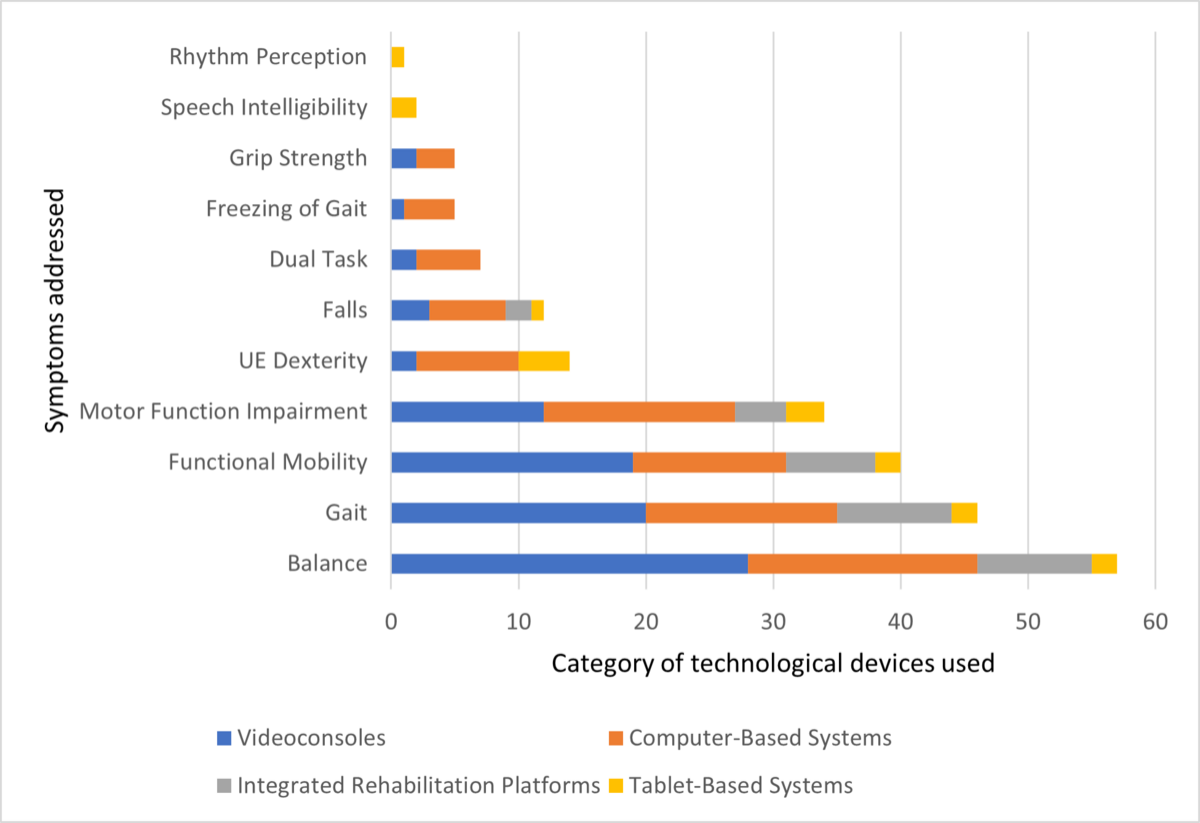
Symptoms addressed by each type of technological device used in rehabilitation. UE: upper extremity.

The technological devices integrating gamification identified across 81 studies were categorized into 4 groups based on the platforms they were used with: video game consoles, computer-based systems, tablet-based systems, and integrated rehabilitation platforms. Detailed information on each category and the specific motor symptoms in PD they address can be found in Figure 2.

##### Video Game Consoles

Video game consoles like the Nintendo Wii and Microsoft Xbox, originally designed for entertainment, were frequently repurposed for rehabilitation. The Nintendo Wii was the most used device, appearing in 27% (22/81) of all studies, almost exclusively in combination with the Wii Balance Board (20/22, 91% of all Nintendo Wii studies). Most studies with this video game console addressed balance (18/22, 82% of studies), gait (12/22, 55%), functional mobility (11/22, 50%), and motor function impairment (9/22, 41%). The Microsoft Xbox, used with the Kinect motion sensor in 14% (11/81) of studies, was similarly focused on balance (10/11, 91% of studies), gait (8/11, 73%), functional mobility (7/11, 73%), and motor function impairment (3/11, 28%). None of the video game consoles were created with rehabilitation as their primary goal. A total of 85% (28/33) of studies using video game consoles delivered their interventions in an in-person format, while 13% (4/33) used telerehabilitation and 3% (1/33) combined both formats.

##### Computer-Based Systems

Computer-based systems were used in 40% (32/81) of studies, often in combination with motion-sensing devices like Kinect (12/32, 37.5% of computer-based systems), Leap Motion Controller (7/32, 22%), and balance boards or force plates (7/32, 22%). Kinect was applied mainly to improve balance (8/12, 67%), gait (7/12, 58%), and motor function impairment (5/12, 42%), while balance boards and force plates focused on balance (7/7, 100%), gait (4/7, 57%), and functional mobility (4/7, 57%), and the Leap Motion Controller targeted upper extremity dexterity (7/7, 100%), motor function impairment (4/7, 57%), and grip strength (3/7, 43%). The HTC Vive Pro VR device was used (2/32, 6%), enhancing immersion for balance (2/2, 100%), gait (2/2, 100%), and functional mobility (2/2, 100%) training. Additionally, some studies combined treadmills with Kinect (3/32, 9%), mainly to focus on falls (3/3, 100%). A total of 91% (29/32) of all computer-based systems were created with rehabilitation as their primary goal, and 78% (25/32) of studies using computer-based systems delivered their interventions in an in-person format, while 22% (7/32) used telerehabilitation.

##### Tablet-Based Systems

Tablet-based systems appeared in 11% (9/81) of all studies, either as standalone devices (6/9, 67% of all tablet-based systems) or combined with motion-sensing devices such as the Leap Motion Controller (1/9, 11%) or handheld tools like GripAble (1/9, 11%). These tablet-based systems mainly targeted upper extremity dexterity (4/9, 44%) and motor function impairment (3/9, 33%). A total of 100% (9/9) of tablet-based systems were created for rehabilitation as their primary goal and 22% (2/2) of tablet-based systems delivered their interventions in an in-person format, while 78% (7/9) used telerehabilitation.

##### Integrated Rehabilitation Platforms

Integrated rehabilitation platforms appeared in 10% (8/81) of all studies, testing systems explicitly designed for comprehensive rehabilitation. These platforms were used to rehabilitate balance (8/8, 100%), gait (8/8, 100%), functional mobility (5/8, 63%), and motor function impairment (2/8, 25%). All integrated rehabilitation platforms were created for rehabilitation as their primary goal. A total of 100% of interventions using integrated rehabilitation platforms were delivered in an in-person format.

### Justification Behind the Use of Technological Devices Integrating Gamification

#### Overview

In order to determine the justifications used by each study for the inclusion of gamification elements and technological devices for motor rehabilitation in PD, four key categories were identified: (1) gamification as a benefit for therapeutic interventions, (2) evidence of effectiveness of technology in rehabilitation for PD, (3) evidence of effectiveness of technology in rehabilitation across populations, and (4) specific properties of technological solutions in rehabilitation.

#### Gamification as a Benefit for Therapeutic Interventions

Gamification was described as a means to support adaptive and individualized rehabilitation [45,47]. Systems were used to monitor user performance, adjust task difficulty based on scoring, and provide real-time feedback throughout the intervention. Some interventions also allowed for the customization of the experience to better match user needs and progression.

#### Evidence of Effectiveness of Technology in Rehabilitation for PD

##### Improvement of Motor Symptoms in PD

Technological devices were frequently described as effective [34,44,47,51,57,61,62,69-71,74-76,79,83,85-87,91,93,96,102,104-106,112-114], promising [38, 48, 52, 64, 67, 68, 79, 81, 88, 102, 111], or feasible [61,72,88,114] tools for addressing motor symptoms in individuals with PD. Studies emphasized their role in improving key functions such as balance, gait, and postural control, while others framed them as having strong potential for rehabilitation, even when not yet fully established through outcomes. Articles also pointed to their feasibility as motor rehabilitation strategies, highlighting aspects like affordability and accessibility to support their use in both clinical and home-based settings.

##### Improvement of Nonmotor Symptoms in PD

Technological devices were described as having a supportive role in addressing cognitive function and emotional well-being in people with PD. Studies reported improvements in areas such as cognition, executive functioning, self-esteem, pain, and mental health [43,48,61,69,71,75,81,87,92,96,97,105,106,109]. Other aspects such as the potential of these technologies to create controlled, structured environments that can enhance the delivery of cognitive therapies and provide emotional support were also highlighted [64,81,100,105].

##### Positive Influence on Brain Activity Networks in PD

Studies discussed the role of technology in supporting underlying neurological processes relevant to rehabilitation [37,40,43,55,58,64,65,69-71,83,94,98,102]. VR and exergames were noted for their potential to promote neuroplasticity, motor learning, and motor-cognitive integration. These interventions were often associated with repetitive, goal-directed tasks designed to engage and optimize brain networks involved in movement and coordination.

### Evidence of Effectiveness of Technology in Rehabilitation Across Populations

#### General Evidence for Physiotherapy

Studies highlighted that technology could offer rehabilitation outcomes comparable to, and in some cases exceeding, those of traditional physiotherapy [39,47,71,77,84,101]. Articles specifically noted the effectiveness of VR and exergames in enhancing therapy for both older adults and individuals with PD, supporting their use as a valid approach across different populations.

#### Evidence for Non-PD Populations

Technology was also supported through evidence drawn from broader populations, including stroke patients and older adults [53,54,74,75,77,91,102,112,113]. In these contexts, VR and video game–based interventions were associated with improvements in key rehabilitation outcomes such as balance, mobility, and fall reduction. These findings were used to justify the applicability of similar technological approaches in the context of PD rehabilitation.

#### Improvement of Clinical Parameters

Some studies pointed to the broader clinical value of technology in rehabilitation, highlighting its role in improving quality of life, physical function, daily activity performance, and pain management [48,51,52,74,86,97]. These outcomes were observed across a range of populations, including children, adults, and individuals with PD.

### Specific Properties of Technological Solutions in Rehabilitation

#### Enhances Motivation Through an Engaging Experience

Technology was described as a tool to make rehabilitation more engaging and enjoyable [36,39,42,43,45,48,50,56,59,60,64,65,67,69-71,83,85,87-89,91,94,96-100,102,103,105,107-109,111,113,114]. By offering interactive and rewarding experiences, these interventions were seen as helpful in encouraging active participation and supporting long-term adherence to therapy programs.

#### Provides Quality Biofeedback

Real-time feedback was noted as a valuable feature in many interventions [37,38,41-45,51,54,55,63,65,66,73-75,77,80,81,83,84,91,92,94,98,100,102,103,106,107,109-112,114]. These feedback mechanisms were associated with enhanced motor learning, improved performance, and greater awareness during rehabilitation tasks.

#### Customization of Training Parameters

The ability to tailor exercises to individual needs emerged as a key strength of technological solutions [39-41,45,48,51,53,57,63,65,70,72,73,76,84,91,94,97,100,101,105,107,111]. Customizable settings enabled repeated practice in varied contexts, supporting motor skill development and encouraging problem-solving during rehabilitation.

#### Enriched or Ecological Environment

Some technologies were highlighted for their capacity to simulate real-world environments, helping users practice tasks in settings that reflect daily life [48,54,55,60,63,72,74,76,78,81,84,92,97,98,101,109,112,113]. These ecologically valid scenarios were viewed as beneficial for promoting skill transfer beyond the therapeutic setting.

#### Supports Dual-Task Integration

Studies mentioned that technological interventions incorporated features that combined physical and cognitive tasks, making it possible to train complex dual-task scenarios [62,66,76,81,83,92,93,95,97,100,106,111]. This was particularly relevant for engaging users both mentally and physically within a single rehabilitation activity.

#### Enables Telerehabilitation

Technology was presented as a practical solution for home-based rehabilitation [36,58,61,83,97,100,103,106]. Devices described as user-friendly and affordable were positioned as a means of extending therapy beyond clinical settings while maintaining effectiveness.

#### Accessible and Cost-Effective

Portability and low cost were mentioned as noted advantages, especially in tools designed for home use [36,61,74,79,82,87,97,104]. These features lead to an easier integration into daily routines, which may contribute to better consistency and accessibility in rehabilitation.

#### Safe Intervention

Studies emphasized the safety of technological interventions, particularly in controlled environments [50,89,98,112,113]. This was considered relevant for tasks that carry a higher risk of falls or injury, offering a secure space for functional improvement.

## Discussion

### Principal Findings

This scoping review aimed to identify and classify the technological devices integrating gamification used in motor rehabilitation for PD, as well as to describe the rationale behind their application. This review is particularly relevant as it addresses a growing interest in leveraging innovative, technology-driven approaches to manage PD’s motor symptoms, a field where gamification has been suggested to enhance patient engagement and therapeutic outcomes [20]. Despite this potential, the integration of gamification into PD rehabilitation interventions remains underexplored, with a limited understanding of how these elements are being applied in practice or their theoretical justifications.

Findings reveal that while technological devices integrating rehabilitation are widely used, the range of gamification mechanics is narrow, with a strong focus on the development and accomplishment core drive. This result aligns with statements by Kai [21], according to which the core drive of development and accomplishment is the easiest to design and the one drawing the most focus from developers. Commonly used elements like feedback mechanisms, points, and progression systems dominate interventions, reflecting an emphasis on monitoring and improving performance. This aligns closely with the most targeted motor symptoms—balance, gait, and functional mobility—which significantly benefit from feedback-driven adjustments offered by motion-sensing technologies, aiding postural control in individuals with PD [117]. Gamification mechanics such as time pressure and consequences from the loss and avoidance core drive are used less frequently with the goal of instilling urgency and accountability, improving speed and efficiency. Elements from this core drive should be carefully considered in designing future interventions, since different motor phenotypes of PD appear to respond differently to reward or punishment-based systems [118]. Similarly, competition mechanics foster motivation and social interaction, aiding long-term engagement. However, more intrinsic motivational pathways, such as those tied to unpredictability and curiosity or ownership and possession, remain underused. Notably absent are complex gamification features like narratives or exploratory elements, highlighting a gap in current approaches.

Out of all the studies included in this review, only 2 [45,47] explicitly refer to the concept or use of gamification when describing their interventions, and none used a theoretical framework to justify their use. Additionally, 37 out of the 81 included studies used technological devices originally designed for entertainment in healthy populations rather than as rehabilitation tools tailored to individuals with health conditions. These findings underline clear research gaps in applying gamification to its full potential in the rehabilitation of people with PD, as well as highlighting an overreliance on video game consoles and other devices designed for entertainment purposes (ie, Nintendo Wii and Microsoft Kinect) rather than rehabilitation-specific technologies. The high presence of interventions based on commercial video consoles and video games for rehabilitation presents an intriguing landscape within this context. On the one hand, leveraging refined, off-the-shelf commercial software offers distinct advantages, eliminating the need for costly development efforts and providing products inherently designed for entertainment, which may enhance participant engagement. However, it is essential to recognize that these interventions lack tailored considerations for populations affected by neurodegenerative conditions, such as PD. Furthermore, the feedback mechanisms embedded in these software options are geared toward game performance rather than exercise and motor learning, potentially rendering them less relevant for the rehabilitation of individuals with PD. As a result, gamification elements, digital environments, and gameplay patterns may not align optimally with the requirements and objectives of this specific population [119]. User-centered design would be an essential part of development in this context, incorporating the needs and preferences of individuals with PD into the interventions [120]. Notably, people with PD exhibit distinct differences from the younger populations typically targeted by commercial video games, which could lead to unintended outcomes when using them to apply rehabilitation interventions [121].

The delivery format of interventions varied by technology type. Video game consoles and computer-based systems were primarily used in in-person settings (85% [28/33] and 78% [25/32], respectively), while tablet-based systems were predominantly used in telerehabilitation (7/9, 78%). Despite most devices being designed for at-home use, researchers tended to implement interventions in supervised settings, likely to ensure safety, adherence, and standardized assessments. This preference aligns with findings suggesting that motion-sensing and immersive rehabilitation technologies often require clinical oversight for effective implementation, particularly in neurodegenerative conditions such as PD [122].

This review identified 4 distinct categories through content analysis of the justifications provided by studies for using technological devices integrating gamification for rehabilitation. Collectively, these categories underscore expectations of such solutions as being safe, motivational, and engaging tools for addressing diverse conditions. They are bolstered by extensive evidence of potential and proven effectiveness and offer innovative training opportunities, including telerehabilitation, dual-task integration, and advanced biofeedback. Despite these well-defined expectations, articles give minimal or no attention to the gamification elements that significantly contribute to their interventions. Current evidence of gaming interventions is unclear, with no studies isolating the effects of gamification used in interventions on people with PD [123]. These findings suggest that while technological solutions show great promise for advancing rehabilitation for individuals with PD, greater emphasis on thoughtfully integrating gamification elements could enhance outcomes by providing more tailored and effective interventions for this population [24].

### Implications for Future Research and Clinical Practice

Many of the interventions in this review relied on commercial entertainment video games, which are not inherently designed for rehabilitation. Their built-in feedback systems and progression mechanics are primarily meant to enhance gameplay rather than support motor learning or therapeutic outcomes for individuals with PD. However, these games remain widely used because they are engaging, cost-effective, and easily accessible. Rather than focusing solely on developing new rehabilitation-specific games, a practical approach could involve evaluating existing commercial games to determine their suitability for therapy. Establishing clear assessment criteria, such as a standardized checklist, could help clinicians identify which games align best with rehabilitation goals and patient needs.

Future research should also explore how well these technologies adapt to the specific challenges faced by individuals with PD, particularly in terms of usability and accessibility. Additionally, a deeper understanding of how different gamification elements influence engagement and adherence, especially when grounded in motivational theories, could help refine rehabilitation strategies. Identifying the most effective elements for sustaining participation and improving outcomes would allow clinicians to make better-informed choices and develop interventions that truly meet the needs of people with PD. To advance this field, research should also prioritize a structured, evidence-based approach to gamification, ensuring that technological solutions are designed with both therapeutic effectiveness and user experience in mind. Integrating User-Centered Design principles with established motivational frameworks could help create interventions that are both engaging and clinically relevant, thus optimizing their impact in neurorehabilitation settings.

### Limitations

This scoping review adhered to the PRISMA-ScR [31] recommendations to ensure methodological rigor. However, several limitations warrant acknowledgment:

Due to the substantial volume of retrieved studies, an initial screening was conducted using only titles and abstracts. This step involved 3 different reviewers who reached a consensus in cases of disagreement. Despite this method, there remains a risk of inadvertently excluding relevant papers.

Another limitation consists of the review’s scope being bound by the search date at which it was performed. Consequently, any studies published after this date were not considered. There is also a limitation in that gray literature, which may contain valuable insights, was not included in the search. As a result, some relevant research might have been overlooked. Articles published in languages other than English and Spanish were also not included, which may also omit relevant information from this review.

This review did not systematically analyze the usability, accessibility, or adaptability of the interventions, as these factors were not part of the predefined data extraction criteria. While these aspects are important for the success of rehabilitation technologies, more research is needed to understand how well gamified interventions address the unique motor and cognitive challenges of individuals with PD.

Finally, there is also a limitation in how the selection of gamification elements for this study was performed. While efforts were made to identify gamification elements in all included studies based on manuscript information and web-based sources, certain custom-made software options and commercial games were inaccessible or had limited available information. Consequently, some gamification elements may not have been fully accounted for.

### Conclusions

This scoping review sheds light on the widespread adoption of technologies integrating gamification elements for motor symptom rehabilitation in individuals with PD. However, it also reveals a notable gap in understanding and acknowledging gamification mechanics among researchers and developers. The current landscape of gamification elements within the reviewed interventions predominantly emphasizes performance-based experiences, focusing on straightforward elements like scoring and feedback. Expectations of technological devices integrating gamification applied in rehabilitation contexts are clear, but there is a notable absence of justification for the specific inclusion of gamification through the application of relevant theories or frameworks, indicating a lack of standardization or rationale for the design of these interventions. Further studies in this field should focus on the importance of user-centered design for the inclusion of gamification mechanics that could modify the experience of users, so as to optimize the development efforts to optimally reach the needs of people with PD. There is also a clear need for the development of studies that report on the effects of gamification itself, which would allow developers to precisely deploy elements according to the expected role of these mechanics within their interventions.

## Supplementary material

10.2196/69433 Multimedia Appendix 1 Search strategy for each database.

10.2196/69433 Multimedia Appendix 2 Details of all included studies.

10.2196/69433 Checklist 1 PRISMA-ScR checklist.

## Abbreviations

PD: Parkinson disease
PRISMA-ScR: Preferred Reporting Items for Systematic Reviews and Meta-Analyses Extension for Scoping Reviews
VR: virtual reality
