# Supplementary material for: Gamification Integration in Technological Devices for Motor Rehabilitation in Parkinson Disease: Scoping Review
Source: JMIR Serious Games. 2025 Jul 4;13:e69433. doi: 10.2196/69433 (PMC12252148; doi:10.2196/69433)
Supplement: Multimedia Appendix 1 [file games-v13-e69433-s001.docx]

# Search strategy for each database

## Medline (OVID)

Ovid MEDLINE® and Epub Ahead of Print, In-Process, In-Data-Review & Other Non-Indexed Citations and Daily and Versions

Search date: 23/11/2023

Results: 708

| **#** | **SEARCH** | **RESULTS** |
| --- | --- | --- |
| 1 | games, recreational/ or video games/ | 7722 |
| 2 | exp Games, Experimental/ | 2622 |
| 3 | gamification/ | 117 |
| 4 | exergaming/ | 171 |
| 5 | exp virtual reality/ | 6099 |
| 6 | exp user-computer interface/ | 39552 |
| 7 | Virtual Reality Exposure Therapy/ | 906 |
| 8 | (gamif* or gameful or gameplay or gamelike or edugam* or scoreboard* or “score board*” or badge* or game or games or videogam* or exergam* or ((virtual or augmented) adj3 realit*) or immersi*).ab,ti. | 105178 |
| 9 | or/1-8 | 143180 |
| 10 | Parkinson Disease/ | 83522 |
| 11 | “parkinson*”.ab,ti. | 146168 |
| 12 | 10 or 11 | 154268 |
| 13 | 9 and 12 | 708 |

## Embase

Search date: 23/11/2023

Results: 895

| **ID** | **Búsqueda** | **Resultados** |
| --- | --- | --- |
| 1 | recreational game'/exp | 7251 |
| 2 | 'game'/exp | 9531 |
| 3 | 'gamification'/de | 517 |
| 4 | 'virtual reality'/exp | 27293 |
| 5 | 'virtual reality head mounted display'/exp | 917 |
| 6 | 'virtual reality system'/exp | 2804 |
| 7 | 'computer interface'/de | 36325 |
| 8 | 'virtual reality exposure therapy'/exp | 987 |
| 9 | gamif*:ab,ti OR gameful:ab,ti OR gameplay:ab,ti OR gamelike:ab,ti OR edugam*:ab,ti OR scoreboard*:ab,ti OR 'score board*':ab,ti OR badge*:ab,ti OR game:ab,ti OR games:ab,ti OR videogam*:ab,ti OR exergam*:ab,ti OR (((virtual OR augmented) NEAR/3 realit*):ab,ti) OR immersi*:ab,ti | 121081 |
| 10 | #1 OR #2 OR #3 OR #4 OR #5 OR #6 OR #7 OR #8 OR #9 | 169656 |
| 11 | 'parkinson disease'/exp | 198961 |
| 12 | parkinson*:ab,ti | 211787 |
| 13 | #11 OR #12 | 251914 |
| 14 | #10 AND #13 | 1284 |
| 15 | #14 NOT ('conference abstract'/it OR 'letter'/it OR 'note'/it) | 895 |

## Cochrane Library

Search date: 23/11/2023

Results: 326

- CDSR: 1
- CENTRAL: 325

| **ID** | **Search** | **Hits** |
| --- | --- | --- |
| 1 | MeSH descriptor: [Games, Recreational] explode all trees | 69 |
| 2 | MeSH descriptor: [Games, Experimental] explode all trees | 159 |
| 3 | MeSH descriptor: [Gamification] explode all trees | 26 |
| 4 | MeSH descriptor: [Exergaming] explode all trees | 58 |
| 5 | MeSH descriptor: [Virtual Reality] explode all trees | 1022 |
| 6 | MeSH descriptor: [User-Computer Interface] explode all trees | 1425 |
| 7 | (gamif* or gameful or gameplay or gamelike or edugam* or scoreboard* or (score NEXT board*) or badge* or game or games or videogam* or exergam* or ((virtual or augmented) NEAR/3 realit*) or immersi*):ti,ab,kw | 16218 |
| 8 | {OR #1-#7} | 17114 |
| 9 | MeSH descriptor: [Parkinson Disease] explode all trees | 6251 |
| 10 | (Parkinson*):ti,ab,kw | 13473 |
| 11 | #9 or #10 | 13473 |
| 12 | #8 and #11 | 326 |

## Scopus

Search date: 23/11/2023

Results: 1230

TITLE-ABS-KEY((gamif* or gameful or gameplay or gamelike or edugam* or scoreboard* or “score board*” or badge* or game or games or videogam* or exergam* or ((virtual or augmented) W/3 realit*) or immersi*) AND (parkinson*))

## Web of Science

Data de cerca: 23/11/2023

Resultats: 1049

| **ID** | **Search** | **Hits** |
| --- | --- | --- |
| 1 | TS=(gamif* or gameful or gameplay or gamelike or edugam* or scoreboard* or “score board*” or badge* or game or games or videogam* or exergam* or ((virtual or augmented) NEAR/3 realit*) or immersi*) | 510593 |
| 2 | TS=(Parkinson*) | 223606 |
| 3 | #2 AND #1 | 1049 |

## Epistemonikos

Search date: 23/11/2023

Results: 0

(title:((gamif* OR gameful OR gameplay OR gamelike OR edugam* OR scoreboard* OR “score board*” OR badge* OR game OR games OR videogam* OR exergam* OR ((virtual OR augmented) AND realit*) OR immersi*) AND Pakinson*) OR abstract:((gamif* OR gameful OR gameplay OR gamelike OR edugam* OR scoreboard* OR “score board*” OR badge* OR badgificification* OR game OR games OR videogam* OR exergam* OR ((virtual OR augmented) AND realit*) OR immersi*) AND Pakinson*))

## PsycINFO

Search date: 23/11/2023

Results: 243

| **Set#** | **Searched for** | **Results** |
| --- | --- | --- |
| S1 | (MAINSUBJECT.EXACT.EXPLODE(“Games”) OR MAINSUBJECT.EXACT(“Virtual Reality Exposure Therapy”) OR MAINSUBJECT.EXACT(“Virtual Reality”) OR MAINSUBJECT.EXACT(“Game Theory”)) OR tiab(gamif* or gameful or gameplay or gamelike or edugam* or scoreboard* or “score board*” or badge* or badgificification* or game or games or videogam* or exergam* or ((virtual or augmented) AND realit*) or immersi*) | 76421 |
| S2 | MAINSUBJECT.EXACT.EXPLODE("Parkinson's Disease") OR tiab(Parkinson*) | 39248 |
| S3 | [S1] AND [S2] | 243 |
